# The competitive esports physiological, affective, and video dataset

**DOI:** 10.1038/s41597-024-04364-z

**Published:** 2025-01-11

**Authors:** Maciej Behnke, Wadim Krzyżaniak, Jan Nowak, Szymon Kupiński, Patrycja Chwiłkowska, Szymon Jęśko Białek, Maciej Kłoskowski, Patryk Maciejewski, Kacper Szymański, Daniël Lakens, Kate Petrova, Jeremy P. Jamieson, James J. Gross

**Affiliations:** 1 Faculty of Psychology and Cognitive Science, Adam Mickiewicz University, Poznan, Poland; 2 Cognitive Neuroscience Center, Adam Mickiewicz University, Poznan, Poland; 3 Network Services Division, Poznan Supercomputing and Networking Center, Poznan, Poland; 4 Human-Technology Interaction, Eindhoven University of Technology, Eindhoven, The Netherlands; 5 Department of Psychology, Stanford University, Stanford, USA; 6 Department of Psychology, University of Rochester, Rochester, USA

**Keywords:** Scientific data, Human behaviour, Emotion, Autonomic nervous system, Stress and resilience

## Abstract

Esports refers to competitive video gaming where individuals compete against each other in organized tournaments for prize money. Here, we present the Competitive Esports Physiological, Affective, and Video (CEPAV) dataset, in which 300 male Counter Strike: Global Offensive gamers participated in a study aimed at optimizing affect during esports tournament^1^. The CEPAV dataset includes (1) physiological data, capturing the player’s cardiovascular responses from before, during, and after over 3000 CS: GO matches; (2) self-reported affective data, detailing the affective states experienced before gameplay; and (3) video data, providing a visual record of 552 in-laboratory gaming sessions. We also collected (affect-related) individual differences measures (e.g., well-being, ill-being) across six weeks in three waves. The self-reported affective data also includes gamers’ natural language descriptions of gaming affective situations. The CEPAV dataset provides a comprehensive resource for researchers and analysts seeking to understand the complex interplay of physiological, affective, and behavioral factors in esports and other performance contexts.

## Background & Summary

Affective science is an interdisciplinary field that draws upon methods and findings from psychology, cognitive science, neuroscience, computer science, biology, and other related fields to understand the complexities of affective phenomena and to determine how they influence human behavior. Multimodal data are needed to thoroughly investigate affective phenomena. Different scientific backgrounds equip researchers with unique skills for data collection. For example, psychologists excel in experimental designs, computer scientists excel in data mining from digital platforms, and biomedical researchers excel in collecting biological samples. Interdisciplinary teams leverage these diverse methodologies to approach research questions by collecting comprehensive multi-modal datasets.

Given the expense associated with assembling comprehensive multimodal datasets, there is growing interest in reusing existing datasets, a practice that remains underutilized due to the scarcity of openly shared raw data. For data reuse, it is particularly important to share the raw data rather than the pre-processed data used for analysis, as raw data are needed in order to fully replicate analyses and harness the full potential of the data. This is particularly critical in fields like affective computing, where developing algorithms capable of understanding and adapting to human emotions relies on access to diverse and detailed descriptors of emotional responses. Such openness and transparency not only enhance the potential for interdisciplinary collaboration but also significantly enrich the resources available for affective science. By providing raw, unprocessed data, researchers enable more nuanced analysis, fostering advancements in understanding the complexities of affective phenomena.

Here, we present the Competitive Esports Physiological, Affective, and Video (CEPAV) dataset^1^. Esports represents a rapidly growing field in which well-trained individuals - gamers - compete using video games. In esports, gamers compete while seated in front of a screen, creating an ideal environment to study affective responses, including emotional experiences and real-time cardiovascular reactions to performance^2–5^. This setting allows for the examination of high-stakes performance with continuous real-time monitoring of affective responses at multiple levels. Using esports as a model allowed us to gain insights into the interplay between emotional states and physiological responses during intense gameplay sessions.

We organized a large-scale study around esports competition^6^. We conducted a three-phase experiment involving a novel Synergistic Mindsets Intervention, compared to a control group focused on brain education^7^, to explore its impact on training and competition performance. This included monitoring participants’ progress over two weeks and their performance in a tournament. A one-month follow-up assessed the intervention’s long-term effects. We recorded gamers’ functioning in the laboratory sessions with continuous, non-invasive measurements of physiological, behavioral, and video data (Stages 1 & 3). Strengths of the CEPAV dataset include:

1. Sample size: 300 participants.
2. Multimodality: participants provided 750 hours of cardiovascular, behavioral, and video data during the laboratory sessions. Furthermore, participants answered multiple questionnaires related to gaming-related affect, well-being and ill-being, and answered multiple open-text questions related to affective gaming situations.
3. Multistage: participants provided data on multiple occasions, namely during two laboratory sessions, daily gaming, daily descriptions of the day, and one-month follow-up after the esports tournaments.
4. Internal/external validity: our study offered a unique blend of internal and external validity through the use of controlled experiments paired with real-world outcomes. It included a thorough evaluation of affective and physiological dynamics and implemented a robust, theory-driven intervention.

Other psychophysiological datasets related to affective manipulations exist (see Tables 1–3), but these datasets are usually related to one of the data types included in CEPAV. We reviewed existing openly available datasets of physiological responses to affective manipulations. Compared to the CEPAV strengths, we found that only four databases included physiological, behavioral, and video data along with individual differences measures^8–11^. Seven databases included data collected on multiple occasions^10,12–17^. Only one database included more participants than the CEPAV^18^ dataset. Finally, we found five datasets that included gaming, including simple labyrinth games^9^, racing games^19,20^ shooter games platform games^19^, FIFA^21^, and League of Legends^16^. Only one dataset included data on participants’ performance^16^, while other datasets used gaming as a situational context for the study.

**Table 1:** Existing Affective Psychophysiological Datasets (Part 1).

Dataset name	Reference	Year	N	Affect reports	# of PNS physiological measures	Electrodermal activity	Cardiac measures)	Skin temperature	Respiration	Blood pressure	# of behavioral measures	Video recordings	Accelerometry	Electromyography	Affect induction procedures	# of visits/days	Gaming	Performance	Personality/individual differences
CEPAV	^1^	2024	300	1	3	0	1	0	0	1	2	1	1	0	Computer Game	2	1	1	Modified Differential Emotions Scale^43^, Regulation of Emotion Systems Survey – Ecological Momentary Assessment (RESS-EMA)^44^, Growth Mindset Scale^45,46^, Stress Mindset Measure^47,48^, Self-Esteem Scale^49^, Body Awareness Questionnaire^50,51^, Satisfaction with Life Scale^52–54^, Flourishing Scale^55,56^, The Self-rated Health Item^57^, Patient Health Questionnaire-2^58^, The Generalized Anxiety Disorder-2^59^, Gaming Disorder Test^60^, Perth Alexithymia Questionnaire^61^, Emotion Beliefs Questionnaire^62^, Appraisal Scale^63^, Negative Appraisals^7^, Demands and Resources Evaluation^7,64–67^,
DEAP	^68^	2011	32	1	6	1	1	1	1	0	1	0	0	1	Music Video	1	0	0	—
MAHNOB-HCI	^69^	2011	27	1	4	1	1	1	1	0	2	1	0	0	Film	1	0	0	—
RECOLA	^70^	2013	46	1	2	1	1	0	0	0	1	1	0	0	Film	1	0	0	—
MAPD	^8^	2014	250	1	3	1	1	0	0	0	1	1	0	0	Film	1	0	0	Eysenck Personality Questionnaire^71^
SWELL-KW	^72^	2014	25	1	2	1	1	0	0	0	3	1	0	0	Presentation Preparation	1	0	0	—

N = number of participants, PNS = peripheral nervous system. For the columns: Affect reports, Electrodermal activity, Cardiac measures (e.g., ECG, PPG), Skin temperature, Respiration, Blood pressure, Video recordings, Accelerometry, Electromyography, Gaming, Performance = 1 represents that these measures were included in the dataset, whereas 0 represents lack of given measure. For the columns:# of PNS physiological measures, # of behavioral measures, and # of visits/days = numbers represent the number of given measures included in the dataset.

**Table 2:** Existing Affective Psychophysiological Datasets (Part 2).

Dataset name	Reference	Year	N	Affect reports	# of PNS physiological measures	Electrodermal activity	Cardiac measures	Skin temperature	Respiration	Blood pressure	# of behavioral measures	Video recordings	Accelerometry	Electromyography	Affect induction procedures	# of visits/days	Gaming	Performance	Personality/individual differences
DECAF	^12^	2015	30	1	1	0	1	0	0	0	3	1	0	1	Film & Music Video	2	0	0	—
BP4D +	^73^	2016	140	1	5	1	1	1	1	1	1	1	0	0	Jokes, Pictures, Videos, Sudden Burst sf Sound, Interview, Silly Song Improvisation, Physical Threat, Cold Pressor, Smelly Odor	1	0	0	—
BioVid Emo DB	^74^	2016	94	1	2	0	1	0	0	0	2	1	0	1	Film	1	0	0	—
ASCERTAIN	^11^	2016	58	1	2	1	1	0	0	0	1	1	0	0	Film	1	0	0	The Big Five Marker Scales^75^
emoFBVP	^14^	2016	10	1	2	0	1	0	1	0	5	1	1	0	Face/Body Manipulation	2	0	0	—
ERiML	^76^	2017	61	1	1	0	1	0	0	0	0	0	0	0	Music/sounds	1	0	0	—
DREAMER	^77^	2017	23	1	1	0	1	0	0	0	0	0	0	0	Film	1	0	0	—
ERS	^78^	2018	50	1	2	0	1	0	0	0	2	0	1	0	Film, Music/sounds	1	0	0	—
AMIGOS	^10^	2018	40	1	2	1	1	0	0	0	1	1	0	0	Film	2	0	0	The Big Five Marker Scales^75^
WESAD	^79^	2018	17	1	5	1	1	1	1	0	2	0	1	1	Reading a story, Film, Trier Social Stress Test	1	0	0	—
CLAS	^80^	2019	62	1	3	1	1	0	0	0	1	0	1	0	Film, Pictures, Math Problems Test, Stroop Test, Logic Problems Test	1	0	0	—
CASE	^81^	2019	30	1	5	1	1	1	1	0	1	0	0	1	Film	1	0	0	—
MPED	^17^	2019	23	1	3	1	1	0	1	0	0	0	0	0	Film	2	0	0	—
Multimodal eSports Dataset	^16^	2020	10	0	3	1	1	1	0	0	5	1	1	1	Computer Games	3	1	1	—
K-EmoCon	^82^	2020	32	1	6	1	1	1	0	0	3	1	1	1	Naturalistic Conversations	1	0	0	—

*N* = number of participants, PNS = peripheral nervous system. For the columns: Affect reports, Electrodermal activity, Cardiac measures (e.g., ECG, PPG), Skin temperature, Respiration, Blood pressure, Video recordings, Accelerometry, Electromyography, Gaming, Performance = 1 represents that these measures were included in the dataset, whereas 0 represents lack of given measure. For the columns:# of PNS physiological measures, # of behavioral measures, and # of visits/days = numbers represent the number of given measures included in the dataset.

**Table 3:** Existing Affective Psychophysiological Datasets (Part 3).

Dataset name	Reference	Year	N	Affect reports	# of PNS physiological measures	Electrodermal activity	Cardiac measures	Skin temperature	Respiration	Blood pressure	# of behavioral measures	Video recordings	Accelerometry	Electromyography	Affect induction procedures	# of visits/days	Gaming	Performance	Personality/individual differences
DAPPER	^15^	2021	142	1	2	1	1	0	0	0	0	0	0	0	—	5	0	0	Big Five Inventory^83,84^, Beck Depression Inventory-II^85^, Self-Esteem Scale^86^, Belief in True Selves^87^, Self-Concept Scale^88^, Meaning in Life Questionnaire^89^, Ten-Item Personality Inventory in China^90,91^
ECSMP	^92^	2021	89	1	4	1	1	1	0	0	1	0	1	0	Film	1	0	0	Emotion Regulation Questionnaire^93^, Self-rating Depression Scale^94^, Profile of Mood States Questionnaire^95^, Pittsburgh Sleep Quality Index^96^
UBFC-Phys	^97^	2021	56	1	4	1	1	1	0	0	1	1	0	0	Speech Preparation, Arithmetic Task	1	0	0	—
DEAR-MULSEMEDIA	^98^	2021	18	1	3	1	1	0	0	0	0	0	0	0	Multiple Sensorial Media	1	0	0	—
AGAIN	^19^	2022	122	1	0	0	0	0	0	0	1	1	0	0	Computer Game	1	1	0	—
BIRAFFE2	^9^	2022	103	1	2	1	1	0	0	0	3	1	1	0	Pictures, Sounds, Computer Game	1	1	0	NEO Five Factor Inventory^99^; The Game Experience Questionnaire (GEQ) Core Module^100^
POPANE	^18^	2022	1157	1	2–6	1	1	1	1	1	0	0	0	0	Speech Preparation, Pictures, Gratitude Text message, Film	1	0	0	—
EMOGNITION	^101^	2022	43	1	4	1	1	1	0	0	3	1	1	0	Film	1	0	0	—
K-EmoPhone	^13^	2023	77	1	3	1	1	1	0	0	1	0	1	0	—	7	0	0	Big Five Inventory^84^, Patient Health Questionnaire^102^, General Health Questionnaire^103^
A2ES	^104^	2023	47	1	2	0	1	0	0	0	0	0	0	0	Film	1	0	0	—
Player experience	^20^	2023	30	1	2	1	1	0	0	0	1	1	0	0	Computer Game	1	1	0	—

*N* = number of participants, PNS = peripheral nervous system. For the columns: Affect reports, Electrodermal activity, Cardiac measures (e.g., ECG, PPG), Skin temperature, Respiration, Blood pressure, Video recordings, Accelerometry, Electromyography, Gaming, Performance = 1 represents that these measures were included in the dataset, whereas 0 represents lack of given measure. For the columns:# of PNS physiological measures, # of behavioral measures, and # of visits/days = numbers represent the number of given measures included in the dataset.

Some information about our study is detailed in the published registered report^6^ - which presents hypothesis testing related to the effects of the Synergistic Mindsets Intervention - including a comprehensive description of the sampling procedures, study procedure, questionnaires, and physiological data. In this paper, we provide additional details on open-text responses, video recordings, and behavioral data. Furthermore, we include new information regarding data quality and present how the measures changed over the course of laboratory visits. Finally, to enhance the usability of the CEPAV dataset, we standardized and merged the physiological and behavioral data collected from three different devices (each with distinct data formats and sampling rates) and uploaded the resulting user-friendly files instead of the raw data.

## Methods

We present data collected in the Psychophysiological Laboratory at the Faculty of Psychology and Cognitive Science, Adam Mickiewicz University, by the International team of researchers for the study aimed at optimizing affective responses during esports tournament^6^ from April 2023 to September 2024 in Poznan, Poland. Additional information on research questions, methods, and analysis are available elsewhere^6^.

### Participants

The sample consisted of 300 male players of Counter-Strike: Global Offensive (CS: GO), aged between 18 and 32 years, with a mean age of 21.95 years (SD = 2.29). Competitive experience varied within the group: 200 players (67%) had no experience, 76 (25%) had competed in local tournaments, 17 (6%) had participated nationally, and six (2%) had taken part in international competitions. One participant did not disclose his competitive background. Esports provided an additional income source for 17 participants, while the rest did not earn money through gaming. On average, participants had been playing CS: GO for 9.13 years (SD = 5.22), with a mean total gameplay time of 2225.69 hours (SD = 1980.55) as recorded in their Steam Library (Valve Corp., SA). The number of participants varied across CS: GO ranks, with 9 participants ranked as Silver I, 2 as Silver III, 7 as Silver IV, 4 as Silver Elite, 4 as Silver Elite Master, 10 as Gold Nova I, 14 as Gold Nova II, 12 as Gold Nova III, 11 as Gold Nova Master, 28 as Master Guardian I, 21 as Master Guardian II, 26 as Master Guardian Elite, 27 as Distinguished Master Guardian, 29 as Legendary Eagle, 32 as Legendary Eagle Master, 19 as Supreme Master First Class, 44 as Global Elite. Details related to inclusion and exclusion criteria and the process of sample size determination are described elsewhere^6^.

### Ethics Information

The study received approval from the Bioethical Committee at Poznan University of Medical Sciences in Poland (802/22). Written informed consent was obtained from all participants, specifying which types of data—self-reports, physiological, behavioral, and video—they agreed to share publicly. This process ensured participants explicitly consented to data publication and the sharing of their images while also allowing them the option to opt out of sharing any specific type of data. For their participation, individuals were compensated with a shopping voucher worth 400 PLN (approximately $100). In addition, esports tournament winners were awarded prizes of 2500, 1500, and 1000 PLN for first, second, and third places, respectively, equivalent to approximately 600, 360, and 240 USD.

### Procedure

**Fig. 1:**
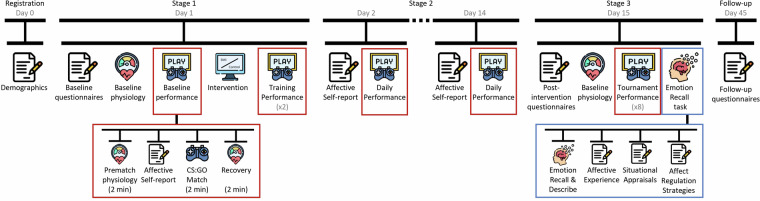
Project and Match Procedures. The red frames represent a procedure for all performances (to simplify the figure, we depicted it in detail only for baseline performance), namely prematch physiology, affective experience, Counter-Strike: Global Offensive match, and recovery. Baseline and post-intervention questionnaires include negative prior mindsets, positive and negative affective experiences, affect regulation strategies, well-being, ill-being, alexithymia, and emotion belief measures. Affective self-report includes affective experience and demands and resources evaluation. Emotion recall tasks include recalling and describing situations from the tournament that elicited positive and negative affective experiences and evaluating them using affective experience, situational appraisals and affect regulation strategies measures. One month after Stage 3, participants were asked to fill in follow-up questionnaires, the same set as at baseline and post-intervention. Figure reproduced from our previous article^6^, used under a CC BY license.

Participants attended two individual laboratory sessions (Fig. 1). During the first session, baseline data and training-related measures were collected (Stage 1). Participants were then randomly assigned to one of two groups: a control group, which learned general facts about the brain, or the Synergistic Mindsets Intervention (SMI) group, which focused on using reappraisal techniques to approach both the performance situation and their responses to it more productively. Further information on the SMI, control interventions, and cover story and transcripts of all instructions given to participants (in Polish and their English translations) were published as the supplementary materials of the hypothesis testing article and can be found elsewhere^22^. Those in the SMI group practiced reappraisal strategies over a two-week period, documenting their adherence and improvements (Stage 2). The follow-up laboratory visit saw participants competing in an esports tournament (Stage 3). We then explored the SMI’s enduring effects with a 1-month follow-up.

#### Stage 1

Upon their first visit to the lab, participants were briefed on the study’s procedures and gave informed consent, specifying which data they were willing to share publicly (for details on how many participants were unwilling for their data to be shared publicly, see Technical Validation/Missing Data section). Next, psychophysiological sensors were attached, and participants completed baseline questionnaires (Tables 4–6). Instructions and questionnaire responses were managed through two 23-inch PC monitors (one dedicated to gaming and the other to experimental tasks). Following the setup and questionnaire completion, the researcher checked the signals quality and moved from the experimental room to the control room, signaling the start of the experiment. The researcher monitored the safety and participant behavior using the camera installed behind the participant.

**Table 4:** Description of Questionnaires Used in the Project (Part 1).

Variable	Questionnaire Name	Citation	Translation	Item Example (Subscale name)	Responses	Examples of Previous Usage	Reliability (Cronbach’s α)		Registration	Stage 1		Stage 2		Stage 3			Follow up
							Previous	Present		Questionnaire Set	Matches	Daily Matches	Daily Reports	Questionnaire Set	Matches	Emotion recall Task	
Positive Affect	Modified Differential Emotions Scale	^43^	Own	*amusement, excitement, joy, and pride*	1 (*strongly disagree*) to 7 (*strongly agree*)	^105–107^	.82-.94	.74-.88		x	x	x	x	x	x	x	x
Negative Affect	Modified Differential Emotions Scale	^43^	Own	*anger, fear, overwhelm, and stress*	1 (*strongly disagree*) to 7 (*strongly agree*)	^105–107^	.82-.94	.74-.88		x	x	x	x	x	x	x	x
Situational Affect Regulation	Regulation of Emotion Systems Survey – EMA	^44^	Own	*I thought of other ways to interpret the situation* (Reappraisal)	1 (*strongly disagree*) to 7 (*strongly agree*)	^44,108–110^	.88-.94	NA		x				x		x	x
Fixed mindset	3-item Growth Mindset Scale	^45^	^46^	*Your intelligence is something about you that you can’t change very much*	1 (*strongly disagree*) to 5 (*strongly agree*)	^7,47,111–113^	.70-.90	.92-.96		x				x			x
Stress mindset	Stress Mindset Measure	^47^	^48^	*The overall effect of stress on my life is negative*.	1 (*strongly disagree*) to 5 (*strongly agree*)	^7,48,111–113^	.80-.91	.78-.88		x				x			x
Self-esteem	Single-Item Self-Esteem Scale	^49^	Own	*I have high self-esteem*.	1 (*strongly disagree*) to 7 (*strongly agree*)	^114–116^	N/A	N/A		x				x			x
Interoception	Body Awareness Questionnaire	^50^	^51^	*I notice distinct body reactions when I am fatigued*.	1 (*strongly disagree*) to 7 (*strongly agree*)	^117,118^	.80-.82	.74-.88		x				x			x
Satisfaction with life	3-item Satisfaction with Life Scale	^52,53^	^54^	*The conditions of my life are excellent*.	1 (*strongly disagree*) to 7 (*strongly agree*)	^119–121^	.86-.94	.70-.83		x				x			x
Flourishing	Flourishing Scale	^55^	^56^	*I lead a purposeful and meaningful life*	1 (*strongly disagree*) to 7 (*strongly agree*)	^122–124^	.80-.89	.79-.88		x				x			x

For columns: Registration, Stage 1–3, Follow up – x represents that given measure was included in that project phase (for the description see Fig. 1).

**Table 5:** Description of Questionnaires Used in the Project (Part 2).

Variable	Questionnaire Name	Citation	Translation	Item Example (Subscale name)	Responses	Examples of Previous Usage	Reliability (Cronbach’s α)		Registration	Stage 1		Stage 2		Stage 3			Follow up
							Previous	Present		Questionnaire Set	Matches	Daily Matches	Daily Reports	Questionnaire Set	Matches	Emotion recall Task	
Health	The self-rated health item	^57^	Own	*In general, would you say your health is?*	1 (*poor*) to 5 (*excellent*)	^125–127^	N/A	N/A		x				x			x
Depressive symptoms	Patient Health Questionnaire-2	^58^	^128^	*Feeling down, depressed, or hopeless”*	0 (*not at all*) to 3 (*nearly every day*)	^129–131^	0.83	0.71-.72		x				x			x
Anxiety symptoms	The Generalized Anxiety Disorder-2	^59^	^132^	*Feeling nervous, anxious, or on edge”*	1 (*not at all*) to 4 (*nearly every day*)	^131–133^	0.81	0.40-.48		x				x			x
Problematic gaming	Gaming Disorder Test	^60^	^134^	*I have had difficulties controlling my gaming activity*.	1 (*Never*) to 5 (*Very Often*)	^60,134^	0.92	0.76-.79	x	x				x			x
Alexithymia	Perth Alexithymia Questionnaire	^61^	Own	*When I’m feeling bad, I can’t tell whether I’m sad, angry, or scared*.	1 (*strongly disagree*) to 7 (*strongly agree*)	^61^	0.82	0.72–81		x				x			x
Emotion Beliefs	Emotion Beliefs Questionnaire	^62^	Own	*People cannot control their NE*. (NEC), *People cannot control their PE* (PEC), *NE are harmful*. (NEU), *PE are harmful* (PEU)	1 (*strongly disagree*) to 7 (*strongly agree*)	^135–137^	0.70-0.88	NEC .71–79, PEC .76–78, NEU .74–79, PEU .51-.67		x				x			x
Intervention Evaluation	Program Feedback Scale	^138^	Own	*I enjoyed the program*.	1 (*strongly disagree*) to 7 (*strongly agree*)	^138–140^	0.88	0.84-0.87			x				x		

NE - negative emotions; PE - positive emotions; NEC - negative emotions’ controllability; PEC - positive emotions’ controllability, PEC; NEU- negative emotions’ usefulness; PEU -positive emotions’ usefulness; For columns: Registration, Stage 1–3, Follow up – x represents that given measure was included in that project phase (for the description see Fig. 1).

**Table 6:** Description of Questionnaires Used in the Project (Part 3).

Variable	Questionnaire Name	Citation	Translation	Item Example (Subscale name)	Responses	Examples of Previous Usage	Reliability (Cronbach’s α)		Registration	Stage 1		Stage 2		Stage 3			Follow up
							Previous	Present		Questionnaire Set	Matches	Daily Matches	Daily Reports	Questionnaire Set	Matches	Emotion recall Task	
Situational Appraisals	Appraisal Scale	^63^	Own	… *I had a sense that this situation mattered to me* (Relevance), *… I had a sense that this situation was potentially desirable for me* (Congruence), … *I had a sense that I was responsible for this situation* (Accountability), … *I had a sense that I did not know how this situation was going to turn out* (Outlook certainty), … *I had a sense that I could change this situation for the better* (Coping potential).	1 (strongly disagree) to 7 (*strongly agree*)	^63^	—	—								x	
Negative appraisals	Four items related to demands and resources	^7^	Own	*Today’s tournament felt like a negative threat to me*.	1 (*strongly disagree*) to 5 (*strongly agree*)	^7^	—	.67							x		
Demands and Resources Evaluation	Two items related to demands and resources	^64–67^	Own	*How demanding do you expect the CS: GO match to be?, How able are you to cope with the demands of the CS: GO match?*	1 (*not at all*) to 6 (*extremely*)	^63–66^	N/A	N/A			x	x			x		
Response Quality Checks	“Directed query” attention and effort checks	^141,142^	Own	*Please select “Strongly agree” for this item to show that you are paying attention*. (directed query); *In your honest opinion, should we use your data in our analyses in this study?* (Effort)	1 (*strongly disagree*) to 7 (*strongly agree*), Yes/No		N/A	N/A		x				x			x

For columns: Registration, Stage 1–3, Follow up – x represents that given measure was included in that project phase (for the description see Fig. 1)

The initial phase of the experiment involved a 5-minute period for establishing physiological baselines, requiring participants to sit quietly. This was followed by the first CS: GO match to assess baseline performance in a format that would mirror the subsequent tournament without any experimental manipulations (baseline match). Post-match, participants were randomly divided into either the SMI or a control group, where they engaged in a 30–40-minute self-guided intervention. Participants were randomly assigned using a random number generator from RandomLists. The assigned condition was linked to the participant number and hardcoded into the intervention presentation software. Upon reviewing the intervention materials, participants were prompted to integrate the newly acquired strategies into their esports training, which involved playing two additional matches (training matches 1 & 2). To conclude Stage 1, instructions were provided on how to log daily measures. Synergistic mindsets participants were advised to consistently apply affect regulation techniques during their gaming sessions over the next two weeks. Control participants were encouraged to incorporate the learned brain facts into their daily gaming practices.

Participants played Counter-Strike: Global Offensive (CS: GO) in a deathmatch mode on the Dust II map, competing against the highest difficulty level of computer-generated opponents (bots) without random weapons. All matches were structured into prematch baseline measurements (2 minutes), gaming (2 minutes), and recovery (2 minutes) (Fig. 1). During all match phases, we collected cardiovascular, behavioral, and video data. Before each match, participants also shared their affective experience and provided demands and resources evaluations.

#### Stage 2

During the period between laboratory visits, participants were instructed to play CS: GO as per their usual gaming routine. On gaming days, they were asked to designate one match to be played in “performance mode”, mirroring tournament conditions. This involved playing a match that included a 2-minute preparation phase and providing affective self-reports prior to the game (see Fig. 1). Post-match, they were asked to log their scores. Additionally, at the day’s end, participants were asked to report their affect and the total time spent gaming that day.

In the first week of this phase, the SMI participants were prompted to use reappraisal before their match to regulate their affective responses. The following week, this prompt was not given to assess if participants would autonomously utilize reappraisal techniques in their daily gaming. The SMI participants also documented their adherence and progress within the affect regulation training by reporting gaming scenarios where they employed reappraisal (also serving as an intervention booster). Control participants noted their gaming situations without any specific affective characteristics. We used the Smartphone Ecological Momentary Assessment 3 (SEMA 3) application for Stage 2 data collection^23^.

#### Stage 3

Two weeks following the initial laboratory session, participants returned to compete in the esports tournament. This session mirrored the setup of Stage 1, starting with physiological sensor attachment, completing questionnaires, and undergoing a 5-minute physiological baseline. Subsequently, participants played eight tournament matches.

Following these matches, they were asked to evaluate the negative appraisals and undertake an emotion recall task. In an emotion recall task, participants were instructed to reflect on, detail, and assess two distinct moments from the tournament: one that elicited positive emotions and another that elicited negative emotions. Participants evaluated these situations on the dimensions of positive and negative affective experience, appraisals, and affect regulation strategies.

The session concluded with a study evaluation, after which participants were debriefed, screened for any suspicions regarding the study’s aims, and compensated. Finally, we identified esports tournament winners, namley players that collected the most points in eight matches (separately, in the SMI and control group). The top three players in each group were awarded prizes of 2500, 1500, and 1000 PLN for first, second, and third places, respectively, equivalent to approximately 600, 360, and 240 USD. We also awarded a single player in each group that scored the most points in a single match, with the award of 500 PLN.

### Measures

We collected six types of measures in this project, namely questionnaires, open-text, other self-reports, video, physiology, and behavior.

#### Questionnaires

We collected data on individual differences at the beginning of Stage 1 and Stage 3 and during the 1-month follow-up. Details related to each questionnaire are presented in Tables 4–6. Additional details on the included questionnaires (e.g., which items measure specific subscales) are included in the “CEPAV_data” spreadsheet^24^ (the “self_reports” sheet).

#### Open-Text answers

Participants were asked to provide open-text answers while learning about the SMI and control intervention, in daily reports, and during the Emotion Recall Task. The full script of Polish and English materials is available elsewhere^6^. The exact instructions for open-text questions are presented in Tables 7–9.

**Table 7:** Description of the Instructions for Open-text Questions (Part 1).

Question Name (study phase)	Exact instructions for open-text questions
EmoEvent_Baseline (Stage 1, SMI)	Think of a time when you were gaming, and you felt stressed or anxious because you were worried you wouldn’t be able to perform at the highest possible level. Please choose a time when your body had a strong stress reaction. This could mean you were nervous and your heart was pounding, or you might have started to sweat, shake, or feel out of breath. Try to choose a gaming situation that you really cared about because we will come back to this example later. This might be a time when you had to compete with other great players or were trying to accomplish an important goal. In the box below, please explain in detail what thoughts you were having and what your body was doing that made you realize you were feeling stress and negative emotions (write 2 to 5 sentences).
EmoEvent_Intervention (Stage 1, SMI)	Think of a time when you were gaming, and you felt stressed or anxious because you were worried you wouldn’t be able to do a good job. Please choose a time when your body had a strong stress reaction. This could mean you were nervous and your heart was pounding, or you might have started to sweat, shake, or feel out of breath. This might be a time when you had to compete with other great players or were trying to accomplish an important goal. In the box below, please explain in detail what thoughts you were having or what your body was doing that made you realize you were feeling negative emotions (write 2 to 5 sentences).
Reap_Used_toOption1 (Stage 1, SMI)	Remind yourself that the struggles and negative feelings when performing won’t last forever because, as you face and work through those struggles your brain is growing smarter, and you learn how to regulate your emotions. In the box below, answer this question: How could you use this kind of strategy again in an upcoming performance? (or what are some initial steps you could imagine yourself taking to do this during an upcoming performance?) For instance, you could mention: a) I will visualize that my brain’s neurons are growing stronger connections while I’m struggling. b) I will think back on times when I used to not know something (maybe when you were a gaming beginner), but over time, I came to learn more about it and get better at it.
Reap_Used_toOption2. (Stage 1, SMI)	Think of the gaming performance as an opportunity for growth, where you can learn about yourself and develop crucial skills. In the box below, answer this question: How could you use this kind of strategy again in an upcoming performance? (or what are some initial steps you could imagine yourself taking to do this during an upcoming performance?). For instance, you could mention: a) I will focus on skills that I’m good at, that I would like to present during this performance. b) I will identify what skills or moves I can train and test in an upcoming game.
Reap_Used_toOption3 (Stage 1, SMI)	Think of your body’s response (heart beating faster, hand sweating, etc.) as designed to help you reach your peak performance level when you’re facing a difficult challenge, like the 1 vs. 3 games. In the box below, answer this question: How could you use this kind of strategy again in an upcoming performance? (or what are some initial steps you could imagine yourself taking to do this during an upcoming performance?). For instance, you could mention: a) If I find myself feeling anxious, I will remind myself that my body’s stress response is there to help you. b) I will remind myself that my body’s response will only get in my way if I spend energy worrying about it. c) If I’m feeling stressed about the performance, I could use my increased alertness to help me stay focused on the task

SMI – Synergistic Mindsets Intervention.

**Table 8:** Description of the Instructions for Open-text Questions (Part 2).

Question Name (study phase)	Exact instructions for open-text questions
Gamer1_missing (Stage 1, SMI)	Below, you can find out more about what other gamers think of our program. Would you add something to their messages? “I’ve always been a “training player” - I was doing great at training, but I couldn’t even show half of my potential at competitions. I blamed stress for everything. I kept thinking about what would happen if I lost, my teammates would think if I messed up, or my palms were extremely sweaty, and everyone could probably see that I was nervous. Now I know that stress is a normal reaction when I care about something, and I can change the thoughts that interfere with my game to help me. People had to fight to survive in the past, and many survived because of their body’s reaction to the stress. If they could do it, so can we. However, what we all have in common besides stress is that we can all try to become better at what is important to us through consistent training. It is important not to get discouraged just because something is difficult. Since it is difficult, it is all the more satisfying to succeed in our chosen dimension, so it is all the more worth the effort and training. Young player, remember that difficult situations are there to make you even better, and your body and mind are on your side and helping you when you think of them that way! “ In the box below, please indicate which elements J.H. did not mention that you think might be most beneficial in teaching new players.
Gamer2_missing (Stage 1, SMI)	Below, you can find out what Filip “Neo” Kubski - professional esports player, Counter Strike legend - thinks of our program. Would you add something to his messages? During the game, stress was always an additional driving force adding to my skills. Over the years, I noticed that stress appeared only in close situations (such as clutch). I must admit that in the daily game, I even miss these emotions. I noticed the physiological stress response - this elevated heart rate and emotions more difficult to regulate - only at the moment after the end of these “key” moments (for example, when I was left alone against several opponents). During the action itself, I had no time to think about anything other than the gameplay itself. I believe esports primarily plays out in the head, so mental support for players is critical. The ability to master and regulate emotions will definitely help stabilize the level of one’s skills and not leave the results to chance. For most of my career, the topic of psychology in esports was not discussed at all. I think that practically none of the professional players in the first ten years of esports had professional psychological help. That’s why I’m glad that this topic is also being addressed by practitioners and researchers who study esports psychology. In the box below, please indicate which elements Neo did not mention that you think might be most beneficial in teaching new players.
Your message (Stage 1, SMI)	In the box below, please write a personal message to a gamer who will be coming to our program. Describe what you could say to help a gamer who finds her/his gaming overwhelming or stressful. You may want to share your own experience and provide her/him with ideas presented in the module. Write 1-2 paragraphs. Next, you will be asked to record it. For instance, you can mention: a) That the brain forms new, stronger connections when it learns from challenges, or b) That the body’s stress response system–your heart rate, your breathing, and so on supports the brain by fueling it with the oxygen it needs to take on hard challenges, or c) That the gaming performance is an opportunity for growth, where you can develop crucial skills and show how good you are. We may choose some of your statements to share with gamers (anonymously) coming to our project. Remember, the more powerful your message is, the more helpful it will be. You don’t have to worry about spelling or grammar – just focus on sharing your thoughts.
Initial_brain_facts (Stage 1, Control)	Before continuing to the program materials, please share your knowledge about how the human brain works. To begin, write one or two things you already know about the brain.
PhGage_what (Stage 1, Control)	One of the first ways that scientists learned about the brain was from people who had brain injuries. The story of Phineas Gage is a famous example. Phineas Gage worked making railroad tracks. One day, there was an accident and a huge railroad spike shot up from the ground. It went through his cheek and skull and into his brain. Phineas Gage didn’t die. He recovered almost fully. But there was one thing that changed. Lots of people have different guesses about what might have changed when they first hear this story. What do you think might have changed about Phineas Gage?

SMI – Synergistic Mindsets Intervention; Control - Control Intervention.

**Table 9:** Description of the Instructions for Open-text Questions (Part 3).

Question Name (study phase)	Exact instructions for open-text questions
Gamer1_missing (Stage 1, Control)	Below, you can find out more about what other gamers think of our program. Would you add something to their messages? “I always knew that our brain has many functions that allow us to function daily. What I didn’t expect, however, was that the brain is so involved when I’m playing! In fact, everything I need to play - seeing the map, hearing my teammates on headphones and speaking to them, moving around the map with the mouse and keyboard, thinking about my next move, anticipating my teammates’ and opponent’s moves - are all made possible by different areas of my brain!”. In the box below, list what information about the brain A.G. did not mention that you think might be most useful in training new players.
Gamer2_missing (Stage 1, Control)	Below, you can find out more about what other gamers think of our program. Would you add something to their messages? “Now I better understand how the brain works. I now know that my eyes are not responsible for my vision and that I know how to go to the local store is due to my temporal lobe. I learned that when I am stressed, my behavior depends on the cooperation of two parts of the nervous system - one responsible for normal functioning and the other responsible for immediate reactions. So the brain is the main tool of all athletes, including esports players. It’s good to know that all this training in shooting accuracy or tactics has a scientific basis. The fact that we become better at something is related to building all these connections. It is motivating to find training strategies to take full advantage of this. On the other hand, it is sad how much brain damage can impair further functioning. Fortunately, thanks to such injuries, scientists are learning more and more about how the brain works and how to treat various diseases.” In the box below, list what information about the brain J.H. did not mention that you think might be most useful in training new players.
Your message (Stage 1, Control)	Today, you learned about three of the four lobes of the brain: the occipital lobe, the parietal lobe, and the temporal lobe. And you learned a little bit about the frontal lobe when you learned about Phineas Gage. In the box below, please write a personal message to a gamer who will be coming to our program. Write, what did you learn today about the brain. Write 1-2 paragraphs. Next, you will be asked to record it. For example, you can write about: a) How people use their brains to do everyday tasks. b) How different parts of our brains do different things. c) How life can be hard when different parts of the brain are damaged. You can include any other facts that you learned or that you already knew. We may choose some of your statements to share with gamers (anonymously) coming to our project. You don’t have to worry about spelling or grammar – just focus on sharing your thoughts.
Your feedback (Stage 1, SMI & Control)	At the beginning of the study, we told you that we will ask for your feedback. Here is the moment where you can write down your thoughts on which parts of the module worked particular well or wasn’t clear enough.
Daily Reports (Stage 2, SMI)	List some of the gaming situations that elicited strong emotions or stress and the way you used Rethinking to make the situation beneficial to you.
Daily Reports (Stage 2, Control)	List some of the gaming situations that happened to you today.
Emotion Recall Task (Stage 3)	Now, we would like you to think of a situation from the tournament when you felt stressed (pleasant) or any other negative (positive) emotions like anger or sadness (joy or excitement). Please choose a time when your body had a strong stress reaction. This could mean you were nervous (happy) and your heart was pounding, or you might have started to sweat, shake, or feel out of breath (had trouble sitting in a place). In the box below, please explain in detail what happened, what thoughts you were having, how you were feeling in your body, and what made you realize you were feeling negative (positive) emotions (write 2 to 5 sentences).

SMI – Synergistic Mindsets Intervention; Control - Control Intervention.

#### Other self-reports

##### Demographics

Participants detailed their highest level of competitive play (ranging from recreational to international), their professional engagement with esports (as a full-time or part-time job or a non-income generating activity), and their weekly gaming duration in hours. Additionally, they reported their in-game ranking (specifically, the top rank achieved in the past year), the total time spent playing the CS: as recorded by the CS: GO game system within the Steam Library (Valve Corp., USA), average weekly hours spent playing against computer-controlled opponents (bots). We also calculated their total gaming hours over the tournament preceding two weeks from their daily logs. Participants also shared their age and Body Mass Index (BMI).

##### Performance

We collected the number of kills, kills’ assists, and deaths and the match scores as determined by the Counter-Strike: Global Offensive scoring system, which factors in the difficulty of the weapon used and the points earned for each enemy bot eliminated. A higher score reflects superior performance. Gamers’ tournament performance was primarily evaluated based on their total score, making it the main performance index. However, other metrics—kills, assists, and deaths—can provide insight into the participant’s strategy. For instance, a high total score paired with a high number of deaths may indicate a risk-taking approach. Analyzing which strategies proved optimal or suited individual gamers could be valuable for esports coaches, helping them tailor training and game plans effectively. In Stage 2, participants were asked to log their daily match scores, simulating the conditions of the upcoming tournament.

#### Video data

##### Video recordings

**Fig. 2:**
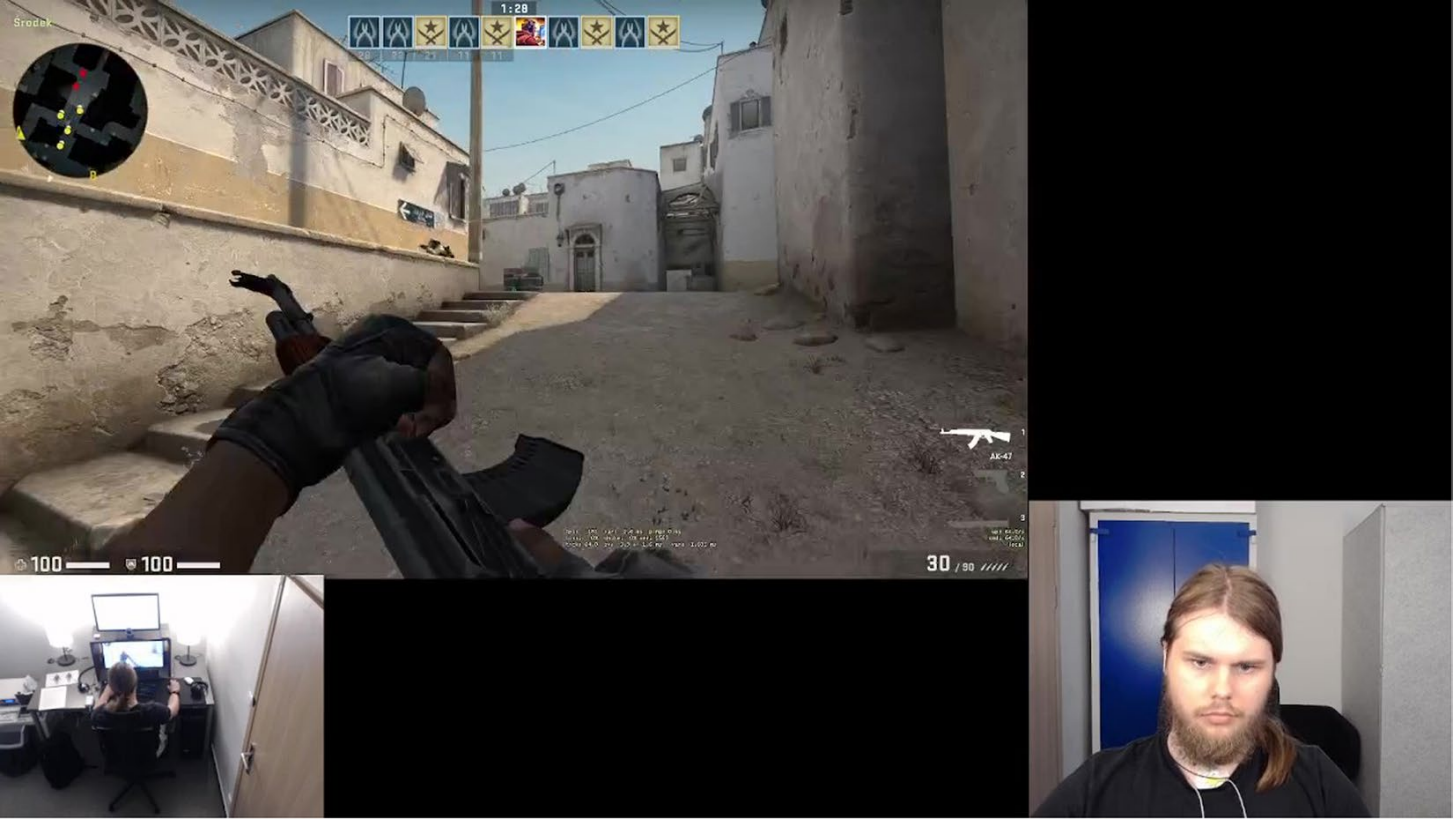
Sample of the Video Data Collected During Stage 1 and Stage 3. The depicted participant provided explicit consent for the open publication of his image.

Participants’ upper bodies were continuously captured on video using an HD camera positioned in between the monitors, utilizing the Open Broadcaster Software^25^. The camera was set approximately 65 cm away from the participants’ heads, with a recording at 30 FPS. We also captured the back view of the experimental view with the camera near the ceiling (Fig. 2). These recordings were primarily used to monitor the study’s progress and ensure participant safety.

##### Gameplay recordings

Gameplay during the CS: GO matches was captured using the Open Broadcaster Software^25^ at 30 FPS to document in-game actions and strategies (Fig. 2).

#### Physiological measures

##### Impedance cardiography and electrocardiography

The Vrije Universiteit Ambulatory Monitoring System (VU-AMS, Netherlands) was employed to collect cardiac biosignals, incorporating both impedance cardiography (ICG) and electrocardiography (ECG) for continuous and noninvasive monitoring of heart activity. In accordance with psychophysiological standards^26,27^, we utilized pre-gelled AgCl electrodes (Kendall Abro, H98SG) positioned according to the standard Lead II setup for ECG measurements and arranged in a four-spot pattern for ICG assessments. This device enables the continuous recording of ECG, Average thorax impedance (ohm; Z0); change in impedance due to respiration and heartbeat (ohm; dZ), and Impedance CardioGram (ohm/s; dZ/dt) to check for proper quality of the recorded signals. Raw data exports were carried out using the VU-AMS Data, Analysis & Management Software (VU-DAMS 5.4.20).

##### Blood pressure

Cardiovascular biosignals were captured using the Finometer MIDI device (Finapres Medical Systems, Netherlands). This device employs the volume-clamp technique with finger cuffs placed on the third digit of the left hand to measure arterial pressure waveforms in the finger, enabling the estimation of Systolic Pressure (mmHg; SBP); Diastolic Pressure (mmHg; DBP); Mean Pressure (mmHg; MAP); Heart rate (bpm; HR); Stroke Volume (ml; SV); Left Ventricular Ejection Time (ms; LVET); Pulse Interval (ms; PI); Maximum Slope (mmHg/s; MS); Cardiac Output (l/min; CO); Total Peripheral Resistance Medical Unit (mmHg.min/l; TPR); Total Peripheral Resistance CGS (dyn.s/cm5; TPRCGS);

#### Behavioral measures

Participant movement was non-invasively tracked using three tri-axial accelerometers (model wGT3X-BT, Actigraph, USA), placed on the thighs (thigh left and thigh right; TL & TR) next to the knee and the right wrist (WR), allowing for the continuous observation of physical activity and gestures during gameplay. All accelerometers were initialized before the participant’s arrival to collect raw acceleration data at 30 Hz with the same start time using ActiLife software (version 6.13.4). We used the measure of the vector magnitude for the given accelerometer for each 1-second interval extracted with the ActiLife software.

### Data preprocessing

Physiological and behavioral data were exported from the acquisition formats by the first author (MB). We used different acquisition software; therefore, the exported data had to be integrated into a common format. The exported TXT and CSV files were preprocessed using Python^28,29^ scientific libraries (e.g., pandas 2.2.2, numpy 1.26.4; see Code Availability, for detailed information). In addition to scripts for processing the typical data, we also added scripts for handling problematic cases and exceptions.

We converted the raw acquired data (obtained with proprietary acquisition software) into a consistent format and saved it in CSV files. All signals were resampled to 1 kHz, using the previous neighbor interpolation method. Signals from different devices were time-synchronized using synchronization markers generated by VU-AMS and Finometer devices during experiments. We marked the baselines, matches, and recoveries within the files. Finally, data across studies were exported to normalized form, consisting of a header, predefined file structure, and standardized subject naming convention. The description of labels used for tagging specific epochs is available in the “CEPAV_data” file^24^, the “epoch_name” sheet.

**Fig. 3:**
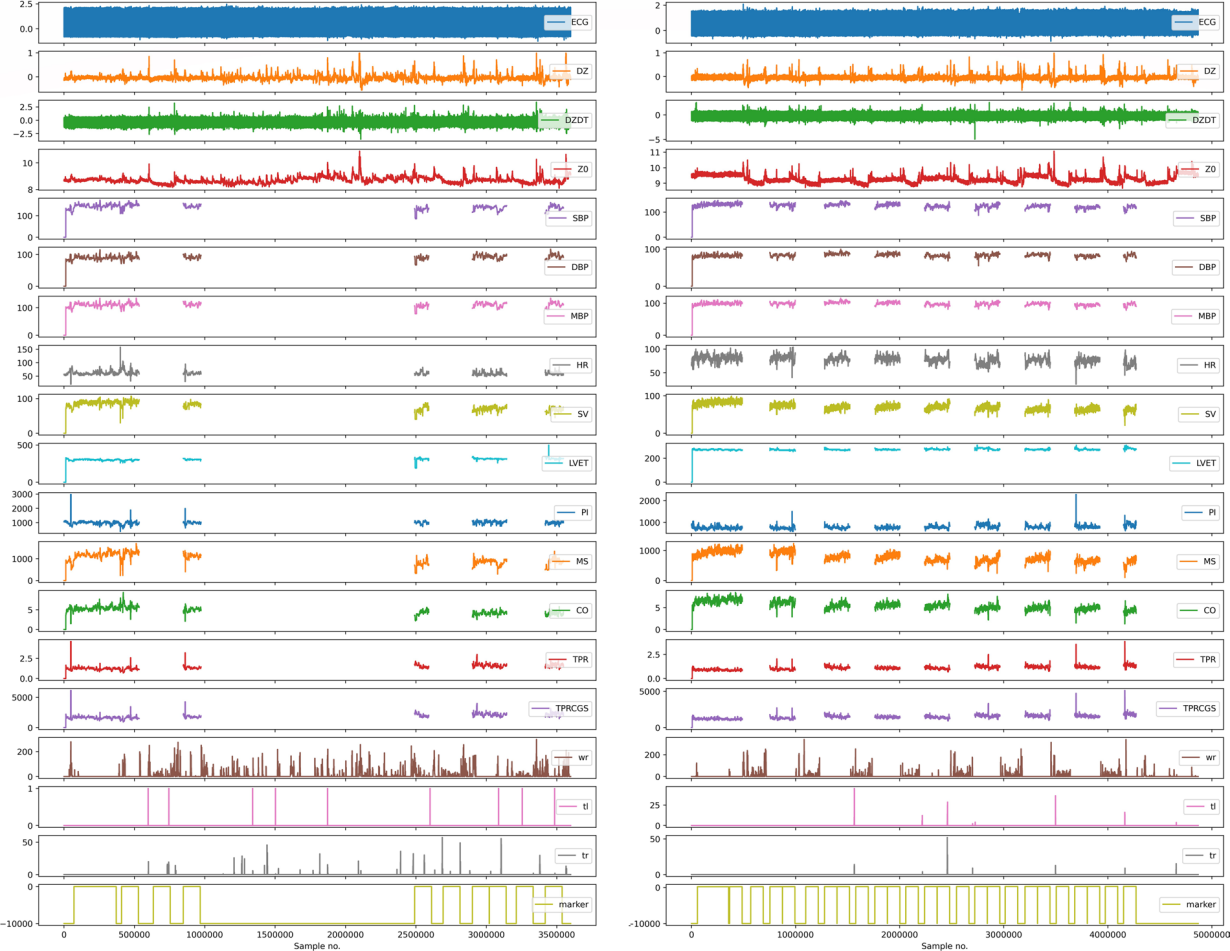
Visualization Single Physiological File Structure for Stage 1 (left panel) and 3 (right panel). ECG- electrocardiogram, mV; Z0 - Average thorax impedance, ohm; dZ - Change in impedance due to respiration and heartbeat, ohm; dZ/dt - Impedance CardioGram, ohm/s; SBP - Systolic Pressure, mmHg; DBP - Diastolic Pressure, mmHg; MAP- Mean Pressure, mmHg; HR - Heart rate, bpm; SV - Stroke Volume, ml; LVET - Left Ventricular Ejection Time, ms; PI- Pulse Interval, ms; MS - Maximum Slope; mmHg/s; CO - Cardiac Output; l/min; TPR - Total Peripheral Resistance Medical Unit, mmHg.min/l; TPRCGS - Total Peripheral Resistance CGS; dyn.s/cm5; wr – right wrist movement, custom units; tl – left thigh movement, custom units; tr – right thigh movement, custom units.

We also prepared examples in Python Jupyter Notebooks presenting how to load and visualize psychophysiological data from sample files (Fig. 3). Both the conversion scripts and the Notebooks can be obtained from our source code repository available at CEPAV Code Component^30^ and at GitHub https://github.com/psychosensing/CEPAV.

For open-text responses, we initially reviewed and corrected any typographical and spelling mistakes. Subsequently, we translated these responses into English using DeepL Translator (DeepL GmbH, Cologne, Germany). Two judges (KS, MB, MK, or SJB) then compared the English translations to the original Polish texts, making adjustments for any clear translation errors. Three judges deliberated on more complex issues and resolved them through consensus.

## Data Records

The CEPAV dataset is publicly available at the *Open Science Framework* (OSF) repository^1^.

### Dataset Structure

#### Self-reports and metadata

We present questionnaires, open-text, other self-reported data, and auxiliary information about the participants in the “CEPAV_data” spreadsheet^24^. The file includes participants’ ID, sex, age, height, weight, experimental conditions, and questionnaire responses (the “self_reports” sheet). To make it easier to use the database, we also included averages for physiological and behavioral data from selected moments of the study in the file, which were used for the *Summary of the Physiological, Affective, and Behavioral Activity During the Competitive Esports* (Technical Validation section) and presented in Fig. 5 (the “physio_behav” sheet)^24^.

#### Video data

The upper-body recordings in an MP4 format, full HD resolution (1920 × 1080) constitute 820 GB of space^31^. The files are grouped into 19 components with a maximum size of 50 GB due to OSF storage restrictions. The component’s name indicates for which study stage (S1 vs S3) and participants and which person they refer to. For instance, the name ‘Video_S3_255_280’ indicates that the component included a video record from Stage 3 for participants from 255 to 280. To ease the video data analysis, we included the time intervals for each experimental epoch in the “CEPAV_data/video_timing” sheet^24^ (e.g., for Stage 1 for Participant 8, the baseline starts at 1:17 minutes of video).

#### Physiological and behavioral data

The physiological and behavioral data, in a CSV format, constitute 366 GB of space^32^. The files are grouped into eight subcomponents with a maximum size of 50 GB due to OSF storage restrictions. The component’s name indicates for which study stage (S1 vs S3) and participants and which person they refer to. For instance, the name ‘Physio_S3_225_300’ indicates that the component included psychophysiological and behavioral data from Stage 3 for participants from 225 to 300. The component contains a set of CSV files for particular subjects. All psychophysiological and behavioral signals recorded during the experiment for each individual are available in a single CSV datafile named “S < stage_id > _P < participant_id >,” where “S” stands for study stage, “P” for participants, e.g., S1_P10.csv, or S3_P224.csv. The “< particpant_id >” is a natural number identifying a participant.

#### Single physiological file structure

Each of the CSV files in the dataset has a 1-line header. The header includes channel/sensor name e.g., “timestamp”, “ECG”, “SBP”, or “marker”. Following the header, each CSV file contains 20 columns. The first column of the data table (except for the header) contains timestamps, as provided by a clock on the main data acquisition (logging) computer – the timestamp shows the time of data collection. In the last column, there is a marker that identifies the specific phase of the experiment. The “epoch_name” sheet provides a full explanation of the epoch IDs used to mark the specific phase of the experiment, e.g., “1” indicates the experimental baseline, while “13” indicates the first minute of the baseline match during Stage 1. The columns in between timestamp and marker contain the physiological data and behavioral data, namely ECG (column 2), DZ (3), DZDT (4), Z0 (5), SBP (6), DBP (7), MAP (8), HR (9), SV (10), LVET (11), PI (12), MS (13), CO (14), TPR (15), TPRCGS (16), WR (17), TL (18), TR (19). For the visualization of a single physiological file structure for Stages 1 and 3, see Fig. 3.

## Technical Validation

### Missing data

The “CEPAV_data” spreadsheet details missing data for various types, organized under the columns: *Missing_ICG_ECG, Missing_Blood_Pressure, Missing_Accelerometry, and Missing_Video* (the “missing” sheet)^24^. Specifically, we were unable to collect some ICG/ECG data from 10 participants, blood pressure data from 10 participants, accelerometry data from 9 participants, and video data from 5 participants. Furthermore, for 7 participants, we deleted some signals that were identified as artifacts during SNR analysis. The spreadsheet also specifies the study stage at which the data is missing; for example, S3_m15 indicates missing data during Stage 3, encompassing tournament performance 5 recovery and tournament performance 6 baseline. Additionally, the absence of ICG and ECG data impacted our ability to compile complete data files for 11 sessions for 10 participants. Furthermore, 22 participants withheld consent to share their video data. None of the participants withheld consent to share their self-reports and behavioral and physiological data.

### Questionnaires reliability

As shown in Tables 4–6, the questionnaires used present acceptable reliability.

### Physiological data - qualitative validation

The physiological data quality was assured by following recommendations in affective science^33^. First, the data were collected by experimenters who completed at least 30 hours of training in psychophysiological research provided by MB. Second, prior to performing preprocessing, the first author (MB) visually inspected all physiological signals. Before inclusion in the database, MB manually double-checked all datasets for missing or corrupted data.

### Physiological data - quantitative validation

We evaluated the quality of the signal with the Signal to Noise Ratio (SNR). In order to calculate SNR across the diverse physiological signals, we used an algorithm based on the autocorrelation function of the signal, using the second-order polynomial for fitting the autocorrelation function curve^34^. We used this approach in our previous project^18^. The script we used for calculating SNR is available in the Code Component^30^ and project’s GitHub repository (https://github.com/psychosensing/CEPAV). The SNR coefficients for all channels for Stages 1 and 3 are available in the “CEPAV_data/SNR” sheet^24^.

The calculated SNR indicated the high quality of all collected signals^35^, with mean SNR ranging from 21.90 dB to 68.05 dB depending on the physiological signal and study. We identified outliers with the median absolute deviation, with a cutoff of 3, as recommended by Leys *et al*.^36,37^, resulting in 416 signals (3.94% of all calculated SNR values) identified as SNR outliers. Next, the first author (MB) visually inspected all flagged data to determine whether the signal should be deleted and classified as an artifact, resulting in 17 signals being identified as artifacts. Outlying signals are marked with yellow color in the “SNR” sheet with additional comments from the first author on reasons that potentially influenced the outlying values (e.g., disconnecting ECG and ICG electrodes after the study while the signals were still being recorded).

**Fig. 4:**
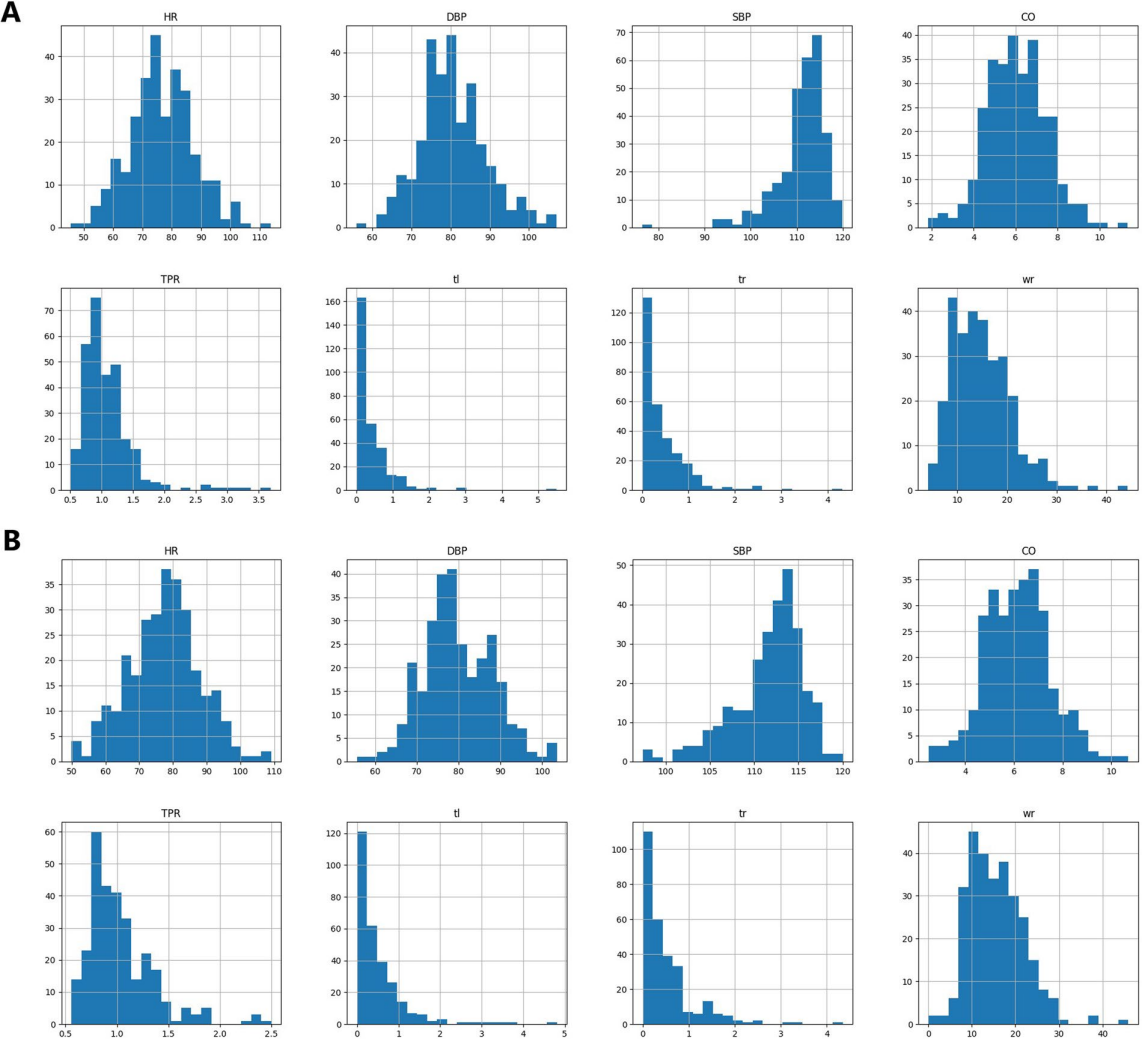
Histograms Presenting Ranges of Means of Collected Signals. Panel A presents data from Stage 1; Panel presents data from Stage 3. HR - Heart rate, bpm, SBP - Systolic Pressure, mmHg; DBP - Diastolic Pressure mmHg; SV - Stroke Volume, ml; LVET - Left Ventricular Ejection Time, ms; PI- Pulse Interval, ms; MS - Maximum Slope; mmHg/s; CO - Cardiac Output; l/min; TPR - Total Peripheral Resistance Medical Unit, mmHg.min/l; TPRCGS - Total Peripheral Resistance CGS; dyn.s/cm5; wr – right wrist movement, custom units; tl – left thigh movement, custom units; tr – right thigh movement, custom units.

We also calculated the mean values of each channel for Stage 1 and Stage 3. Histograms (Fig. 4) present that collected signals had standard ranges. For instance, most participants presented a healthy SBP and DBP range during the experiments^38^. Figure 4 presents data for easily interpretable measures (e.g., HR, SBP, DBP collected with Finometer) and does not present raw recordings (e.g., ECG in mV) that require further processing (e.g., heart rate based on peak analysis).

### Summary of previously completed analyses

In our initial publication, we tested the effects of the Synergistic Mindsets Intervention (SMI) compared to a control intervention^6^. The SMI was positively received, leading participants to adopt more advantageous stress mindsets, more favourable appraisals of the esports tournament, and an increased application of reappraisal strategies for emotion regulation. Despite these positive outcomes, the high-stakes nature of the esports competition was perceived as an enjoyable challenge rather than a negative stressor, reducing the potential for the SMI to significantly influence affective and physiological reactions. The absence of a negative physiological stress response meant there was very little for the intervention to modulate. Consequently, no significant changes were noted in affective responses or gaming performance due to the intervention. Access to the research code, dataset, and findings can be found elsewhere^6^.

### Summary of the physiological, affective, and behavioral activity during the competitive esports

**Fig. 5:**
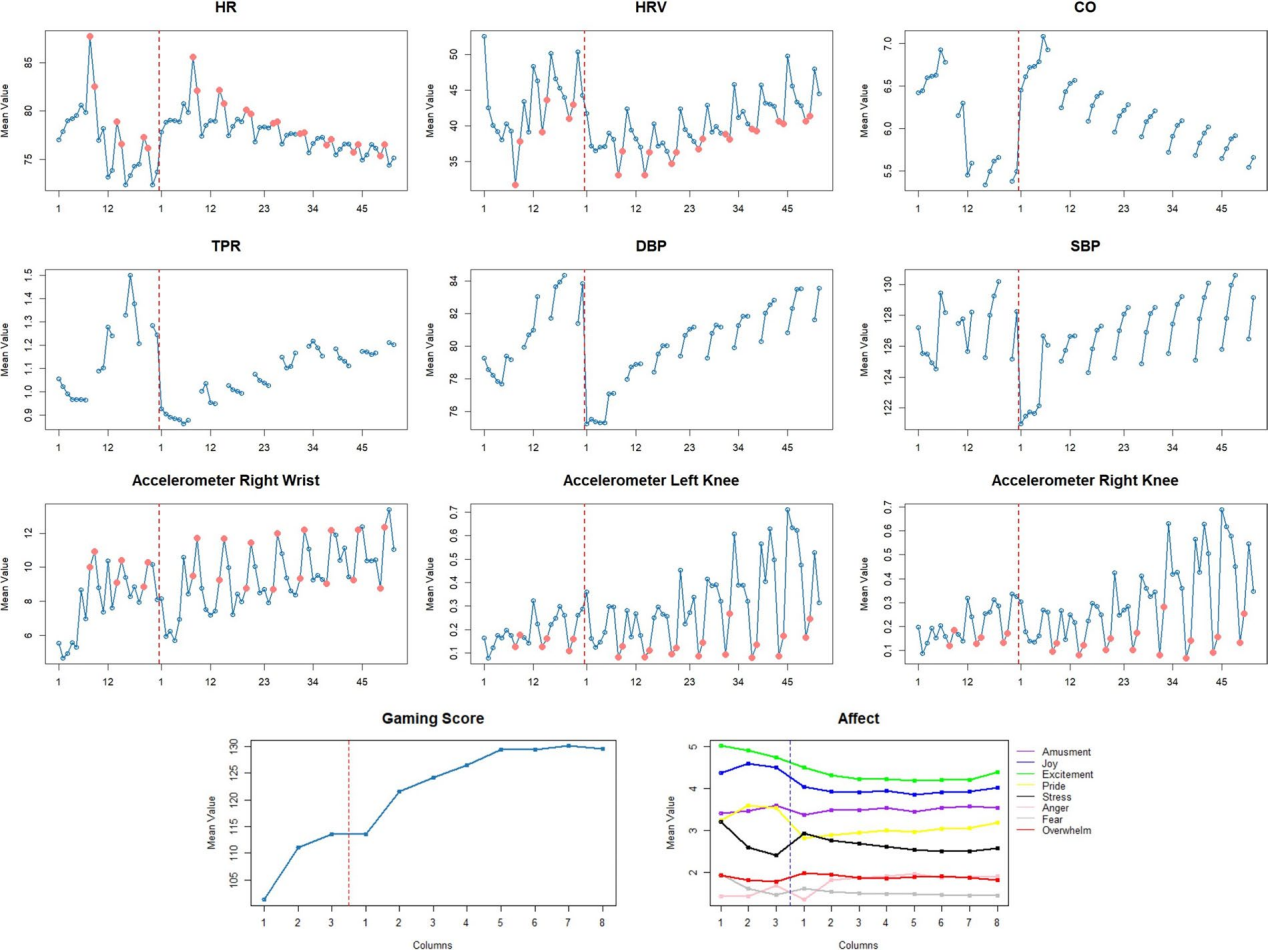
Shifts in Mean Levels of Affective Measures. Red line separates Stage 1 and Stage 3 laboratory visits. HR - Heart rate, bpm, SBP - Systolic Pressure, mmHg; DBP - Diastolic Pressure mmHg; CO - Cardiac Output; l/min; TPR - Total Peripheral Resistance Medical Unit, mmHg.min/l.

To describe competitive gaming from the affective, physiological, and behavioral perspectives, we calculated the averages for the one-minute intervals for given situations within the study: five minutes of resting baseline, pre-match baselines, during the matches, and recoveries after the matches. Figure 5 visualizes the levels of the given parameters: HR, HRV, SBP, DBP, CO, TPR, WR, TL, and TR. As shown, there are clearly visible differences between gaming and resting periods for HR, HRV, and accelerometry. Furthermore, we identified the time-related drift in HR, which may indicate the healthy habituation process^39,40^. Our observations may serve as an inspiration for the quantitative analysis of the observed phenomena.

## Usage Notes

The CEPAV dataset is available at OSF^1^. This dataset holds potential value for a broad range of fields within affective science, including psychology, for exploring the connections between self-reported individual differences and physiological responses; computer science, particularly in the areas of machine learning for the development of automated affect detection and the clustering of gaming-related data; physics, as a practical dataset for examining the technicalities of signal processing; and mathematics, for the verification of mathematical or statistical models. The dataset can also be used to explore methods for performance optimization and examine factors related to gamers’ well-being and ill-being. To simplify the use of the CEPAV dataset, we also recorded two videos in which we discuss 1) the motivation, procedures, and results of the initial study^6^, and 2) the structure of the CEPAV dataset. Both videos are available on the OSF^1^. As the dataset is published under CC-By Attribution 4.0 International license, we hope that CEPAV will provide individuals, companies, and laboratories with the data they need to perform their analyses to advance affective science.

However, the dataset comes with certain limitations. First, this dataset cannot be employed to investigate differences between sexes, ethnicities, or between the group ages, as all participants were male Caucasian young adults. Second, our investigation was confined to affective reactions within a single esports (Counter Strike: Global Offensive) context. Third, as noted in the missing data section, the dataset lacks some data due to technical constraints (e.g., ICG missing due to electrode detachment), lack of consent to share data, and human errors (e.g., not starting data collection for accelerometers). Lastly, the dataset represents a secondary use of data initially collected for a previously published independent study.

In conclusion, the CEPAV database offers a rich and detailed source of data shared with the scientific community to support research and development in the field of affective science. One of the principles of open science is that researchers are free to use the dataset without the need to contact the authors as long as their usage complies with the applicable licenses. However, we encourage researchers to reach out to us at macbehnke@gmail.com if they have any questions or require assistance. We are happy to provide guidance and support to help maximize the utility of the dataset.

### Declaration of Generative AI and AI-assisted technologies in the writing process

During the preparation of this work, the authors used Grammarly and ChatGPT4.0 to improve the readability and language. After using this tool/service, the authors reviewed and edited the content as needed and take full responsibility for the publication’s content.

## Data Availability

The code can be accessed via the *Code* component^30^ on OSF and via the public GitHub repository: https://github.com/psychosensing/CEPAV. It is licensed under MIT OpenSource license, i.e., the permission is granted, free of charge, to obtain a copy of this software and associated files (e.g., the Jupyter IPython Notebooks), subject to the following conditions: the copyright notice and the MIT license permission notice shall be included in all copies or substantial portions of the software based on the scripts we published. Scripts for converting data from proprietary acquisition software formats into consistent CSV files, as well as IPython Jupyter Notebooks presenting how to load the data from CEPAV CSV files into Python Pandas DataFrame structure, are available in the *Code* component^30^ on OSF and at the following GitHub repository: https://github.com/psychosensing/CEPAV. Scripts that we used to transform the data from proprietary acquisition formats into coherent CSV files utilized Python 3.10.12^41^. The list of the specific modules and their versions is available in the “requirements.txt” file in the GitHub repository. Jupyter Notebooks use Python version: 3.10.12, as well as the following Python modules: packages related to Jupyter Notebook: jupyterlab v. 4.0.8; jupyter-core module v. 5.5.0, jupyter-client v. 8.6.0; jupyter_server: 2.10.0, Nbclient v. 0.9.0, nbconvert v. 7.11.0, nbformat v. 5.9.2, traitlets v. 5.13.0ipython v. 8.17.2; ipykernel v. 6.26.0^42^; and a data organization and manipulation module – pandas v. 2.2.2^28^.
